# Supplementary material for: Central Angiotensin II type 1 receptor deficiency alleviates renal fibrosis by reducing sympathetic nerve discharge in nephrotoxic folic acid–induced chronic kidney disease
Source: PeerJ. 2024 Sep 26;12:e18166. doi: 10.7717/peerj.18166 (PMC11439387; doi:10.7717/peerj.18166)
Supplement: Supplemental Information 1 [file peerj-12-18166-s001.docx]

**Supplemental information**

**Central AT1 receptor deficiency alleviates renal fibrosis by reducing sympathetic nerve discharge in nephrotoxic folic acid–induced CKD**

Qijun Wan^1,2*^, Zhichen Yang^3*^, Lingzhi Li^1,2^, Liling Wu^1,2^

**This PDF file includes:**

Supplemental Tables 1

Supplemental Figures 1

**Table S1 Primers used for Real-time PCR**

| **Gene** | **Forward (5’->3’)** | **Reverse (5’->3’)** |
| --- | --- | --- |
| Agtr1a | CTACCGCCCCTCAGATAAC | AGCCATTTAGTCCGATGCT |
| Agt | GGAACGACCTCCTGACTTGG | TCAGATTTGCCTCCGCACC |
| Col1a1 | CAGAGGCGAAGGCAACA | GTCCAAGGGAGCCACATC |
| Gapdh | TGTTTCCTCGTCCCGTAGA | ATCTCCACTTTGCCACTGC |

**
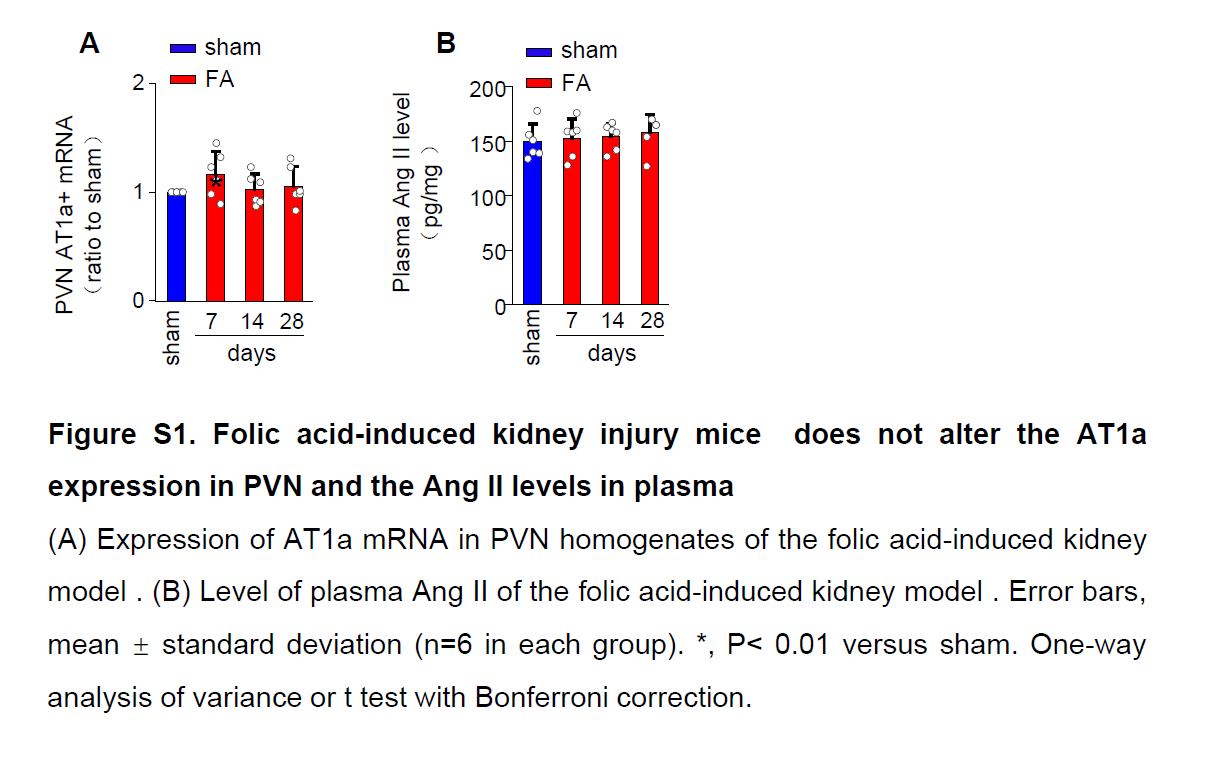
**
